# Heat-induced longevity in budding yeast requires respiratory metabolism and glutathione recycling

**DOI:** 10.18632/aging.101560

**Published:** 2018-09-17

**Authors:** Marina Musa, Matea Perić, Peter Bou Dib, Sandra Sobočanec, Ana Šarić, Anita Lovrić, Marina Rudan, Andrea Nikolić, Ira Milosević, Kristian Vlahoviček, Nuno Raimundo, Anita Kriško

**Affiliations:** 1 Mediterranean Institute for Life Sciences, Split, Croatia; 2 University Medical Center Göttingen, Institute of Cellular Biochemistry, Göttingen, Germany; 3 Ruđer Bošković Institute, Division of Molecular Medicine, Zagreb, Croatia; 4 European Neuroscience Institute, University Medical Center Göttingen, Göttingen, Germany; 5 University of Zagreb, Faculty of Natural Sciences and Mathematics, Zagreb, Croatia; * Equal contribution

**Keywords:** aging, hormesis, heat shock, budding yeast, mitochondria

## Abstract

Heat-induced hormesis is a well-known conserved phenomenon in aging, traditionally attributed to the benefits conferred by increased amounts of heat shock (HS) proteins. Here we find that the key event for the HS-induced lifespan extension in budding yeast is the switch from glycolysis to respiratory metabolism. The resulting increase in reactive oxygen species activates the antioxidant response, supported by the redirection of glucose from glycolysis to the pentose phosphate pathway, increasing the production of NADPH. This sequence of events culminates in replicative lifespan (RLS) extension, implying decreased mortality per generation that persists even after the HS has finished. We found that switching to respiratory metabolism, and particularly the consequent increase in glutathione levels, were essential for the observed RLS extension. These results draw the focus away solely from the HS response and demonstrate that the antioxidant response has a key role in heat-induced hormesis. Our findings underscore the importance of the changes in cellular metabolic activity for heat-induced longevity in budding yeast.

## Introduction

While in the laboratory setting the growth conditions are strictly maintained, cells and organisms in the wild are subject to a wide range of different environmental conditions, from changing temperature to inconsistent nutrient availability. Some of these conditions can be lethal if severe or prolonged, but a vast majority promotes an adaptive response when transient and sufficiently mild. This dose response is known as hormesis. The adaptability of cells was crucial during evolution, as development of complex mechanisms was key to survival in changing environmental conditions. Some of the stressors, like iron and oxygen, were eventually incorporated into the cellular pathways due to their chemical properties [1,2].

In recent years, the adaptive stress response has been more extensively studied in the context of aging and disease progression, as many hormetic treatments have been shown to improve cell fitness and survival, presumably through stimulation of maintenance and repair pathways. The stress response depends on the nature of the stressor and the downstream targets involved [3–5]. Moreover, it has been observed that low levels of one stressor can protect against more than one type of stress. For example, low levels of oxidative stress protect against subsequent exposure to toxins such as cyanide, thus making hormesis “cross-modal” [6,7].

Furthermore, heat, irradiation, and oxidative stress have all been shown to extend lifespan in yeast, fruit fly, nematodes, and rodents [8–12], suggesting that similar pathways are involved in regulation of longevity across organisms [12]. Hormetic effects of heat shock on lifespan extension have been associated with increased transcription of chaperone coding genes, as well as with activation of autophagy and opening of the nuclear envelope [8,11–15].

Given its “cross-modal” nature as well as the variety of triggers and cell responses elicited, hormesis can be considered as a complex phenomenon. Thus, although extensively studied [8,12], the exact mechanisms contributing to heat shock-mediated lifespan extension in yeast still remain to be uncovered. Here, we found that a metabolic switch from glycolysis to aerobic respiration, and the resulting increase in the reactive oxygen species (ROS) levels trigger the activation of the pentose phosphate pathway (PPP) in budding yeast *Saccharomyces cerevisiae* cells undergoing mild HS. We further show that this change in metabolism restores the disrupted redox homeostasis during heat shock by providing NADPH required for glutathione recycling. Enhanced respiration, as well as active glutathione recycling during heat shock is essential for the heat shock-induced replicative lifespan extension.

Taking into account the first observations that yeast lifespan is subject to environmental fluctuations [16] as well as the conserved nature of hormesis, further insights into hormesis-induced yeast longevity may prove invaluable to research of lifespan regulation in higher organisms and to development of mimetics of hormetic agents.

## RESULTS

### Mild transient heat shock causes metabolic reprogramming and lifespan extension

**Figure 1:**
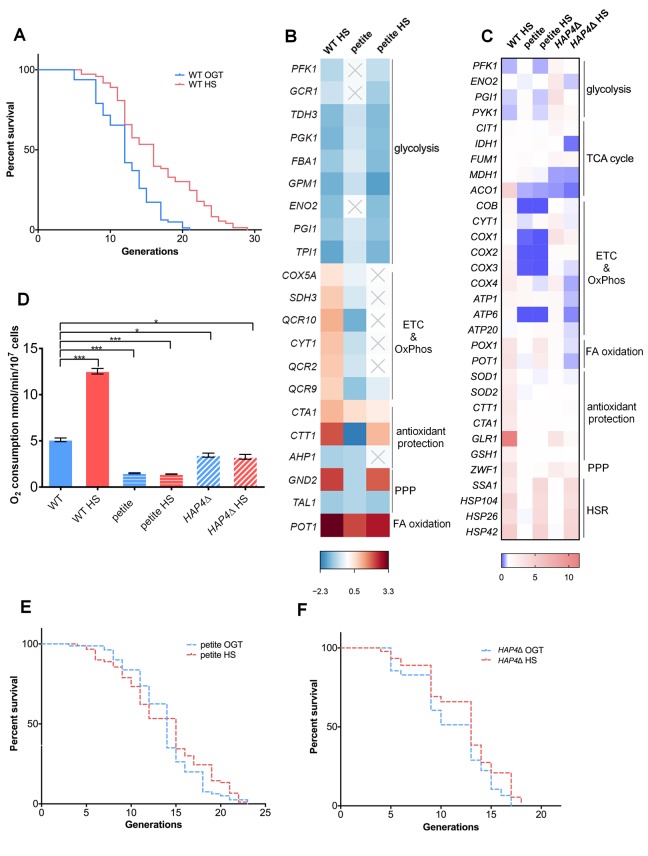
**Mild transient heat shock leads to replicative lifespan extension triggered by enhanced respiratory activity.** (**A**) Mean and maximum replicative lifespan (RLS) is extended in yeast that undergo a 3-hour heat shock at 34^o^C early in their life (1-3 generations). Control cells were kept constantly at 30^o^C. The number of curated cells is 81 for WT and 73 for WT HS. Significance of the results was tested with log-rank (Mantel–Cox) test. *P* value is <0.0001. (**B**) Differential gene expression measured by RNA sequencing (RNA-Seq) reveals changes in cellular metabolic activity, most prominently repression of glycolysis and activation of respiration. Heat map displays RNA-Seq-derived differential expression (contrasted to control) of selected genes, grouped according to their respective metabolic pathways. Gray X signs indicate statistically insignificant fold changes. Gene names are indicated on the left of each heat map. (**C**) qPCR measurement of differential gene expression confirms repression of glycolysis and activation of respiration, as well as reveals activation of the pentose phosphate pathway and glutathione recycling. Color of the squares on the heat map corresponds to the mean value of the log fold change from three biological and three technical replicates. *UBC6* was used for normalization. (**D**) Oxygen consumption is increased at heat shock in WT yeast, but remains unchanged in the respiration deficient petite strain and in *HAP4*Δ strains. Oxygen uptake was measured polarographically using an oxygraph equipped with a Clark-type electrode. Data on the graph are mean ± SEM from three biological and three technical replicates. *P* values were calculated using ANOVA plus post hoc. ***P < 0.001; **P < 0.01; *P < 0.05. (**E**) Mean and maximum replicative lifespan (RLS) remain unchanged in petite strain under heat shock. Control cells were kept constantly at 30^o^C. The number of curated cells is 80 for petite and 89 for petite HS. *P* value is >0.05 (Mantel-Cox). (**F**) Mean and maximum replicative lifespan (RLS) remain unchanged in *HAP4*Δ strain under heat shock. Control cells were kept constantly at 30^o^C. The number of curated cells is 76 for *HAP4*Δ and 92 for *HAP4*Δ HS. *P* value is >0.05 (Mantel-Cox).

Budding yeast *S. cerevisiae* go through a limited number of cell divisions before the onset of senescence, producing daughter cells, the number of which constitutes their replicative lifespan (RLS). In addition to genetic manipulations, RLS can be modulated by environmental stressors such as heat or oxidative stress [8,17,18]. Here, we measured the RLS of yeast mother cells exposed to a mild and transient heat shock (HS) at 34°C. The heat exposure started at the mother cells replicative age of 1-3 generations and lasted for 3 hours, after which they were returned to optimal growth temperature of 30^o^C (for details see Methods). Control cells were kept at 30^o^C for the duration of the experiment. As we were interested in the effect of transient temperature shift, RLS was measured by removing and counting daughter cells continuously until the mother stopped dividing, without refrigerating the plates overnight, in order to avoid introduction of confounding variables. We show that the 3 hour exposure to 34^o^C increases median RLS by approximately 50% (from app. 12 to 18 generations), and the maximum RLS by 38% (from app. 21 to 29 generations), thus supporting previous reports (Figure 1A) [8,12].

Next, we aimed to explore the ensemble of changes that arise in response to the applied HS. To this end, we performed RNA sequencing (RNA-Seq) on yeast cells collected immediately after the 3 hour HS in batch culture, which started during the early exponential growth stage (at OD ∼0.3), to mimic as closely as possible the conditions of the RLS experiment. The most prominent changes observed following HS were related to remodeling of the metabolic activity: repression of genes encoding glycolytic enzymes, upregulation of respiratory chain subunits, as well as upregulation of peroxisomal fatty acid beta-oxidation enzymes (WT HS in Figure 1B, Table S1). Although yeast cells typically rely on glycolysis when glucose is present (exponential growth phase) [19], our results suggest that glycolysis was repressed by HS, and the cells switched to respiratory metabolism despite the presence of glucose.

Such switch in metabolic activity is usually characteristic of diauxic shift, which follows exponential growth and occurs when glucose is exhausted from the medium. Since the utilization of glucose through respiration is characterized by lower flux, the diauxic shift is typically accompanied by increased generation time i.e. slowed growth [19]. However, growth curves in the applied conditions (see Methods for details) show that the division time was actually shortened; the generation time of the WT strain at optimal growth temperature (OGT) was approximately 120 minutes ± 6 minutes, while during HS it decreased to 72 minutes ± 4 minutes. While the origin of this acceleration of division remains unclear, it provides sufficient evidence that the cells did not undergo a diauxic shift due to the exposure to HS (Figure S1).

Transcript level measurements by quantitative real-time polymerase chain reaction (qPCR) confirmed the trend in gene expression changes found by RNA-Seq, as well as revealed some new ones (some genes did not pass the significance cutoff in the RNA-Seq data analysis) (WT HS in Figure 1C). Expectedly, qPCR measurement showed upregulation of *SSA1* (Hsp70p), *Hsp104*, *Hsp26* and *HSP42*, typical of the HS response activation (Figure 1C), validating the employed approach. Curiously, WT cells under HS displayed increased expression of the pentose phosphate pathway (PPP) rate-limiting enzyme glucose-6-phosphate dehydrogenase (G6PD, gene name *ZWF1*), indicating an increase in the PPP activity (Figure 1C). However, only the oxidative branch of the PPP was activated, as evidenced by downregulation of transaldolase (*TAL1*), an enzyme of the non-oxidative branch (Figure 1B). The upregulation of glutathione reductase (*GLR1*) as well as of reactive oxygen species (ROS) scavenging enzymes, superoxide dismutases (*SOD1* and *SOD2*) and catalases (*CTT1* and *CTA1*) (Figure 1C) strongly suggests that the cells were undergoing oxidative stress, even during mild HS. The response consists of concerted upregulation of antioxidant enzymes, as well as investing in redox homeostasis by activation of the PPP, potentially serving as a source of NADPH, an electron donor to several antioxidant reactions, such as reduction of oxidized glutathione (GSSG to GSH).

### Increased respiration is required for RLS extension upon heat shock exposure

As the response to HS was characterized by stark changes in transcript levels of genes encoding respiratory chain subunits, we measured oxygen consumption in the WT strain during HS. After 3 hours of exposure to 34^o^C, oxygen consumption increased 2.5-fold in WT strain (Figure 1D). Motivated by this result, as well as by our previous observations on the role of respiration in the RLS extension [20], we hypothesized that respiration increase may be a requisite for the HS-induced RLS extension. To test this, we measured the RLS of the respiration-deficient yeast strain (petite) in the same conditions as described for WT strain. The petite strain displayed no HS-induced RLS extension: the median RLS was 14 generations at OGT compared to 15 generations after the transient HS exposure, while the maximum RLS was 23 generations in both cases (Figure 1E). The absence of HS-induced RLS extension is consistent with the report of Shama et al. under similar conditions [8]. RNA-Seq analysis (Figure 1B) and the qPCR transcript level measurements (Figure 1C) show that the petite strain metabolic response to HS was similar to the one of WT: glycolysis enzymes underwent a decrease in expression levels, while fatty acid oxidation was activated. The upregulation of the HS proteins Hsp104p, Hsp70p, Hsp42p and Hsp26p demonstrated the activation of the HS response. However, several key features differentiate the response to HS between the petite strain and the WT. Expectedly, the petite strain displayed very low transcript levels of mitochondrial DNA (mtDNA)-encoded respiratory chain complexes (*COB*, *CYT1*, *COX1*, *COX2*, *COX3*), as well as impaired oxygen consumption at optimal temperature, both of which were unaffected by HS (Figure 1C; Figure 1D). Importantly, G6PD or *GLR1* were not upregulated after HS in the petite strain, and neither were antioxidant protection elements (catalases and superoxide dismutases), suggesting that the petite strain did not experience oxidative stress during HS, as opposed to WT (Figure 1C). At this point, we hypothesized that the reason for this difference in gene expression in the petite strain was the absence of enhanced respiratory activity at HS.

In order to further support the role of respiration activation in HS-induced RLS extension, we tested a strain lacking Hap4p (*HAP4*), the transcription factor in charge of upregulating nucleus-encoded components of the respiratory chain [17]. As the only regulatory subunit of the Hap2/3/4/5 DNA binding complex, Hap4p plays a major role in converting yeast cells from fermentative to respiratory growth during the diauxic shift, regulating the expression of genes encoding for respiratory chain subunits and TCA cycle enzymes [21–23]. Hap4p deficiency is, therefore, sufficient to prevent the observed switch to respiratory growth during HS. Unlike the petite strain, *HAP4*Δ strain did respire at OGT (Figure 1D), but failed to increase respiratory activity during the 3 hour HS (Figure 1D). Already at OGT, in the *HAP4*Δ background, the cells displayed increased expression levels of glycolytic enzymes, and decreased levels of TCA cycle enzymes, which were the most prominent differences compared to the WT (Figure 1C). At HS, the transcript levels of glycolytic (*PFK1, ENO2, PGI1*) and fatty acid oxidation enzymes (*POX1, POT1*) underwent downregulation relative to the same strain at OGT, while the TCA cycle enzymes (*CIT1, FUM1, MDH1, ACO1*) mostly remained unaffected, with the exception of *IDH1* which was strongly downregulated, thus likely affecting the overall activity of the TCA cycle (Figure 1C). The transcript levels of the respiratory chain complexes in the *HAP4*Δ background at OGT displayed a modest upregulation compared to the WT (Figure 1C). However, at HS the respiratory chain complexes were downregulated (*COB, CYT1, COX3, COX4, ATP1, ATP6, ATP20*), or remained unaffected (*COX1, COX2*), along with unchanged G6PD and *GLR1* transcript levels (Figure 1C). Consistent with the other strains, the HS response was activated in *HAP4*Δ background, evidenced by the increased expression levels of Hsf1p target genes (*HSP104, SSA1, HSP42* and *HSP26*) (Figure 1C).

Finally, there was no RLS extension following HS in *HAP4*Δ strain; median lifespan at both OGT and HS was approximately 13 generations, with maximum RLS of 17 and 18 generations, respectively (Figure 1F). *HAP4*Δ strain displayed a shortened RLS, consistent with previous reports [24]. With the HS response activated, the absence of the RLS extension in *HAP4*Δ background added to the body of evidence that increased respiratory activity during HS is a major contributor to HS mediated RLS extension and diminishes the relevance of the HSR alone for the mild heat-induced longevity. At this point, we hypothesized that enhanced respiration is acting as a source of increased mitochondrial superoxide levels, triggering the PPP activation, thus enabling glutathione recycling, and likely culminating in RLS extension. We set out to test this sequence of events.

### Increased mitochondrial superoxide levels during heat shock activate the pentose phosphate pathway

**Figure 2:**
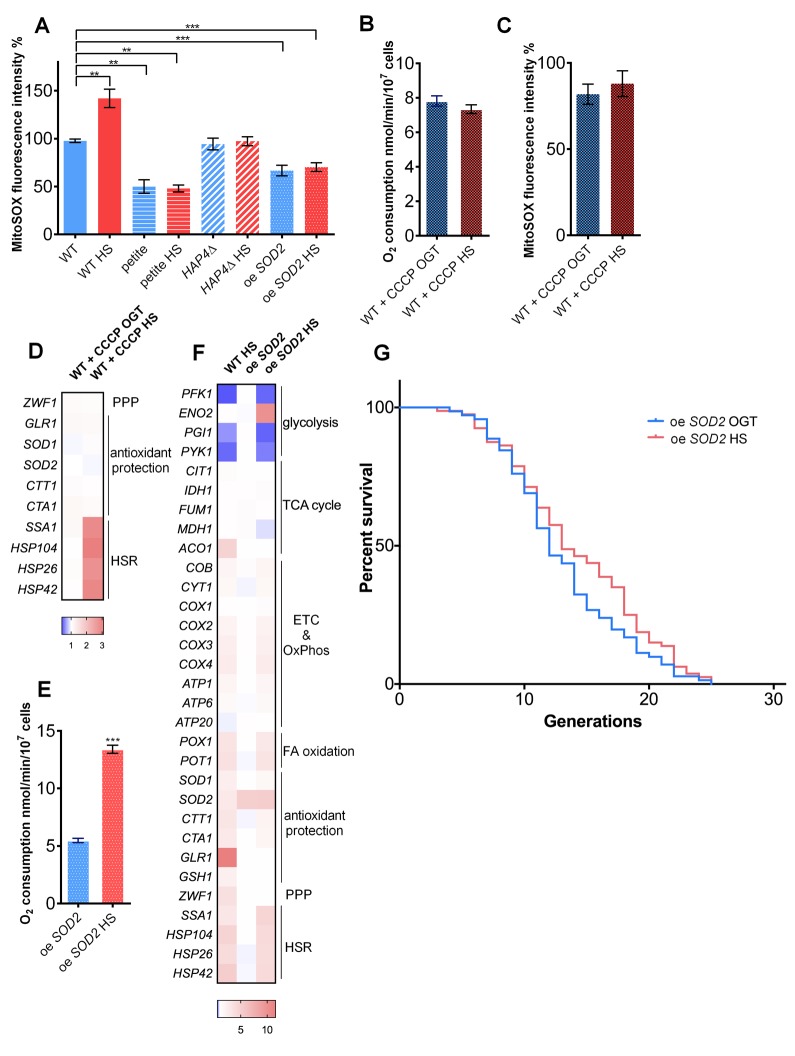
**Increased mitochondrial superoxide levels during heat shock activate the pentose phosphate pathway and redox homeostasis maintenance.** (**A**) Mitochondrially produced superoxide (measured by FACS as MitoSOX fluorescence in 10000 cells) increased during HS in WT, but not in petite, *HAP4*Δ, or oe *SOD2* strains. The results are presented as the percentage of MitoSOX fluorescence detected in WT strain at optimal growth temperature. (**B**) Oxygen consumption increased at optimal growth temperature in the presence of 10 μM CCCP compared with untreated control and remained the same during heat shock. Oxygen uptake was measured polarographically using a Clark-type electrode equipped oxygraph. (**C**) Mitochondrially produced superoxide (measured by FACS as MitoSOX fluorescence in 10000 cells) decreased in the presence of 10 μM CCCP at optimal growth temperature and was unaffected by heat shock. The results are presented as the percentage of MitoSOX fluorescence detected in WT strain at optimal growth temperature. (**D**) qPCR measurement of differential gene expression in the presence of mitochondrial uncoupler CCCP (10 μM) showed that respiration activity is required for the activation of the pentose phosphate pathway and redox maintenance during heat shock. Color of the squares on the heat map corresponds to the mean value of the log fold change from three biological and three technical replicates. *UBC6* was used for normalization. (**E**) oe *SOD2* strain displayed increased respiratory activity at HS. (**F**) qPCR analysis of the oe *SOD2* strain shows increased levels of *SOD2* at both OGT and HS, but no activation of other oxidative stress response enzymes, including G6PD following HS. (**G**) Mean and maximum replicative lifespan (RLS) remain unchanged in oe *SOD2* strain under heat shock. Control cells were kept constantly at 30^o^C. The number of curated cells is 71 for oe *SOD2* and 80 for oe *SOD2* HS. *P* value is >0.05 (Mantel-Cox). Unless otherwise stated, data in graphs are mean ± SEM from three biological and three technical replicates. ***P < 0.001; **P < 0.01; *P < 0.05 (ANOVA plus post hoc).

Thus far, we have demonstrated that enhanced respiratory activity during HS is needed for the HS-induced RLS extension. Increased oxygen consumption in WT during HS (Figure 1D) was accompanied by an approximately 50% increase in the level of superoxide (measured via MitoSOX fluorescence), as well as a 30% increase in mitochondrial volume (Figure 2A; Figure S2). Conversely, petite and *HAP4*Δ strains experienced no significant change in mitochondrial superoxide levels during HS (Figure 2A), consistent with the absence of oxygen consumption increase in the same conditions (Figure 1D). To further support this observation, we measured 2′,7′‐dichlorodihydrofluorescein (H2DCF) fluorescence, which is proportional to intracellular ROS, using a FACS‐based procedure. Consistent with MitoSOX, the H2DCF fluorescence showed an approximately 75% increase in intracellular ROS in the WT strain during HS (Figure S3). Such trend was absent in the petite and *HAP4*Δ strains, consistent with MitoSOX results (Figure S3).

These results support the qPCR data, which showed increased transcript levels of antioxidant enzymes, *ZWF1* and *GLR1* in the WT cells, but not in the petite or in the *HAP4*-deficient strain (Figure 1C). These trends strongly suggested that the activation of the PPP in the WT cells is a part of the antioxidant defense triggered by the increased superoxide levels during HS exposure [25] and demonstrated that in the absence of oxygen consumption increase in petite and *HAP4*Δ strains during HS the cells do not experience oxidative stress.

To additionally test this scenario, we treated the WT cells with carbonyl cyanide *m*-chlorophenyl hydrazone (CCCP), an uncoupler of the respiratory chain, at OGT as well as during HS. The oxygen consumption was increased after addition of CCCP compared to untreated control and remained unchanged during HS (Figure 2B). Similarly, the superoxide levels, which were below those of WT at OGT, remained unchanged after HS in the presence of CCCP (Figure 2C). Moreover, the transcript levels of *ZWF1, GLR1*, catalases, and superoxide dismutases remained at the levels observed at the OGT (Figure 2D). Finally, the heat shock response was activated in the presence of CCCP (Figure 2D). These results suggest a relationship between the excess of superoxide produced during heat shock and the induction of the pentose phosphate pathway, as well as glutathione recycling.

To confirm that the increased levels of mitochondrial superoxide play a key role in triggering the adaptive response to heat shock, we overexpressed the mitochondrial manganese superoxide dismutase (Sod2p, further referred to as oe *SOD2*). The level of overexpression was approximately 7-fold at transcript level, as determined by qPCR (Figure S4). In optimal growth conditions, as well as during HS, the level of mitochondrial superoxide (as measured by MitoSOX) was reduced to average of 65% of the level detected in the WT strain at OGT (Figure 2A), despite the enhanced respiratory activity at HS (Figure 2E). The transcript levels of *ZWF1* (G6PD) failed to increase at HS, indicating that pentose phosphate pathway was not activated (Figure 2F). *GLR1* transcript levels also remained unchanged during HS in the oe *SOD2* strain (Figure 2F). Finally, the oe *SOD2* strain did not display a HS-induced RLS extension: median RLS of the strain at OGT was 12 generations, compared to 13 generation with the HS exposure, with maximum RLS of 25 generations for both conditions (Figure 2G).

These results supported our hypothesis that during HS exposure, the superoxide produced by the respiratory chain triggered the antioxidant defenses, redox homeostasis maintenance, and the PPP activation. We propose that the purpose of the PPP activation is to provide NADPH required for the glutathione recycling, and we turn to test this hypothesis.

### Increased glutathione levels are essential for the heat-induced lifespan extension

**Figure 3:**
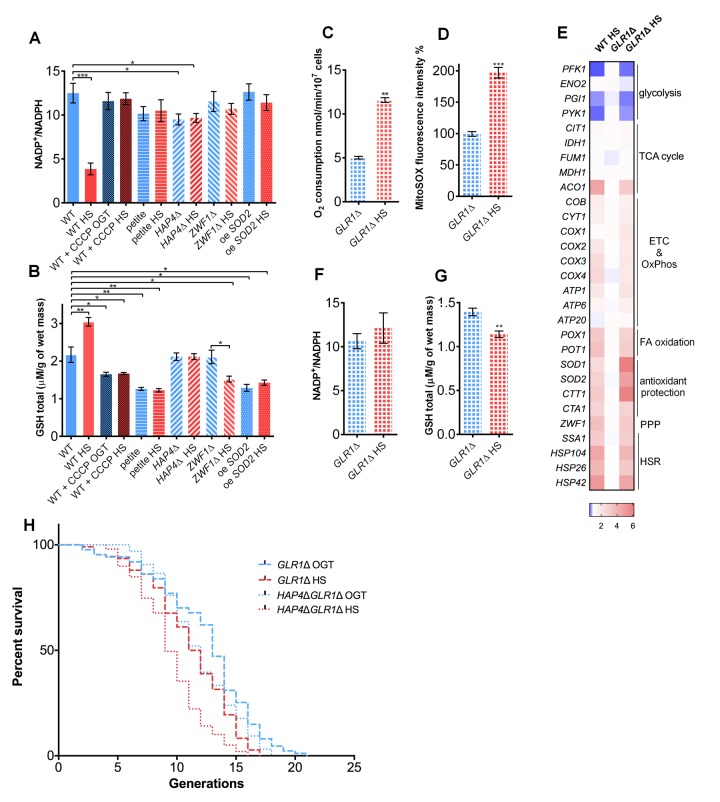
**Glutathione recycling is essential for the heat shock induced replicative lifespan extension.** (**A**) NADP^+^/NADPH ratio decreased in the WT during heat shock, suggesting increased NADPH levels. However, the ratio was unaffected by heat shock in the petite strain, the *HAP4*Δ strain, in the presence of CCCP, in the strain deficient in G6PD (*ZWF1*Δ), or in the strain carrying overexpression of *SOD2*. (**B**) GSH levels were increased in the WT during heat shock. Heat shock did not induce the GSH levels in the petite, *HAP4*Δ strain, WT in the presence of CCCP, in the strain deficient in G6PD (*ZWF1*Δ), or in the strain carrying overexpression of *SOD2*. (**C**) Oxygen consumption was increased under heat shock in the *GLR1*Δ strain. Oxygen uptake was measured polarographically using a Clark-type electrode equipped oxygraph. (**D**) Mitochondrially produced superoxide (measured by FACS as MitoSOX fluorescence in 10000 cells) was increased in the *GLR1*Δ strain during heat shock. The results are presented as the percentage of MitoSOX fluorescence detected in WT strain at optimal growth temperature. (**E**) qPCR measurement of gene expression levels showed that the absence of *GLR1* did not affect the heat shock induced metabolic changes, also observed in the WT. Color of the squares on the heat map corresponds to the mean value of the log fold change from three biological and three technical replicates. *UBC6* was used for normalization. (**F**) NADP^+^/NADPH ratio remained unchanged during heat shock in the *GLR1*Δ strain. (**G**) GSH levels decreased during heat shock in the *GLR1*Δ strain. (H) Mean and maximum replicative lifespan (RLS) were decreased in *GLR1*Δ following heat shock. RLS of the double mutant *HAP4*Δ*GLR1*Δ revealed that *GLR1* is epistatic to *HAP4* under the conditions of mild heat shock. The number of curated cells if 87 for *GLR1*Δ, 107 for *GLR1*Δ HS, 96 for *HAP4*ΔG*LR1*Δ and 99 for *HAP4*Δ*GLR1*Δ HS. *P* value is <0.0001 (Mantel-Cox). Unless otherwise stated, data in graphs are mean ± SEM from three biological and three technical replicates. ***P < 0.001; **P < 0.01; *P < 0.05 (ANOVA plus post hoc).

To further explore the role of NADP^+^/NADPH ratio in the studied strains, we measured it at OGT and after 3 hours of HS. The results showed a decreased NADP^+^/NADPH ratio in the WT strain during HS, however without a change in total NADP levels (NADP_total_, see Methods for details), suggesting increased NADPH levels (Figure 3A, Figure S5). Such trend was not observed in the petite, the *HAP4*Δ, CCCP treated WT strain, or in the oe *SOD2* strain (Figure 3A). Moreover, the decline in the NADP^+^/NADPH ratio observed in the WT at HS was abolished in the G6PD-deficient strain (*ZWF1*Δ), confirming the essential role of the PPP activity (Figure 3A).

One of the cellular roles of NADPH is to contribute to the prevention of oxidative stress by providing electrons to maintain the redox buffers in the reduced form. The most abundant redox buffer in the cell is glutathione (GSH), whose oxidized form (GSSH) is reduced to GSH by NADPH via glutathione reductase (Glr1p) (26). In WT, GSH level increased by approximately 30% during HS (Figure 3B), in line with the increase in the transcript levels of *GLR1* (Figure 1C). However, addition of CCCP to the medium during HS prevented the increase in GSH levels (Figure 3B) or the expression level of *GLR1* (Figure 1C; Figure 2D). Moreover, in the absence of *ZWF1* (G6PD), GSH levels failed to increase, confirming that NADPH is required for glutathione recycling (Figure 3B, Figure S3). In the oe *SOD2* strain, the basal levels of GSH were lower than in WT, and were unperturbed by HS (Figure 3B), most likely because the superoxide was efficiently neutralized by the Sod2p and further antioxidative protection was not required. Based on these results, we hypothesized that the GSH increase may be among the most distal events responsible for the RLS extension in WT at HS. Therefore, we turned to a mutant deficient in Glr1p (*GLR1*Δ), which catalyzes glutathione reduction during HS, thus replenishing the cellular stocks of GSH.

During HS, the oxygen consumption increased in the *GLR1*Δ background (Figure 3C), as did the mitochondrial superoxide levels (Figure 3D), similar to the trends observed in the WT strain (Figure 1D, Figure 2A). Importantly, the qPCR gene expression level measurement revealed that the *GLR1*Δ strain experiences similar changes during HS as the WT strain: the repression of glycolysis, activation of respiration and pentose phosphate pathway are most prominent changes (Figure 3E). The *GLR1*Δ strain also displayed no change in NADP^+^/NADPH ratio at HS, presumably due to accumulation of the NADPH produced by the activated PPP (Figure 3F). Despite the increased availability of NADPH (Figure S5), the GSH levels were significantly lower in *GLR1*Δ at HS (Figure 3G), clearly due to the absence of *GLR1*. Importantly, the HS-induced RLS extension was absent in *GLR1*Δ background; maximum RLS was 19 generations following HS, compared to 21 generations at OGT (Figure 3H, dashed line).

To consider the existence of epistasis between *HAP4* and *GLR1*, we performed RLS measurement on a strain carrying deletions of both *HAP4* and *GLR1* genes, *HAP4*Δ*GLR1*Δ. At optimal growth temperature the RLS of the *HAP4*Δ*GLR1*Δ mutant was consistent with the RLS of *HAP4*Δ with median RLS of 12 generations, and the maximum RLS of 18 generations (Figure 3H, dotted line). Following the exposure to transient HS, the median RLS of the *HAP4*Δ*GLR1*Δ mutant was approximately 10 generations and the maximum 16 generations (Figure 3H, dotted line). The HS-induced RLS extension was absent in the *HAP4*Δ*GLR1*Δ mutant, and the RLS was similar to the one of the *GLR1*Δ strain. Based on this result we propose that during the employed HS *GLR1* is epistatic to *HAP4*, supporting the claim that the enhanced respiration lead to the activation of glutathione recycling. Moreover, these results strongly suggest that mitochondrial superoxide or related reactive oxygen species (ROS) like hydrogen peroxide could be responsible for triggering the activation of the PPP, thus supporting the increase in GSH levels by producing NADPH, ultimately essential for the HS-induced RLS extension in yeast.

### Replenishment of the GSH level during HS relies on Yap1p

**Figure 4:**
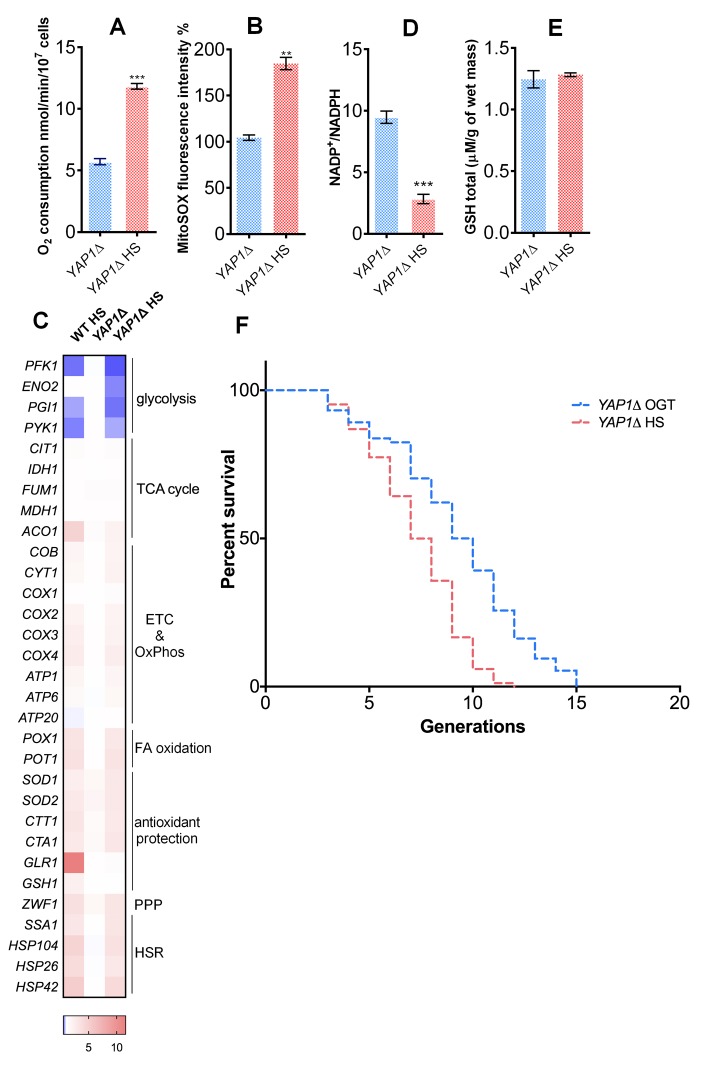
**Yap1p is required for the mild heat shock induced RLS extension.** (**A**) Oxygen consumption was increased under heat shock in the *YAP1*Δ strain. Oxygen uptake was measured polarographically using a Clark-type electrode equipped oxygraph. (**B**) Mitochondrially produced superoxide (measured by FACS as MitoSOX fluorescence in 10000 cells) was increased in the *YAP1*Δ strain during heat shock. The results are presented as the percentage of MitoSOX fluorescence detected in WT strain at optimal growth temperature. (**C**) qPCR measurement of gene expression levels showed that the absence of *YAP1* did not affect the heat shock induced metabolic changes observed in WT. (**D**) NADP^+^/NADPH ratio was decreased during heat shock in the *YAP1*Δ strain. (**E**) GSH levels were not affected by heat shock in the *YAP1*Δ strain. (**F**) Mean and maximum replicative lifespan (RLS) were decreased in *YAP1*Δ already at OGT, and further decreased following heat shock. The number of curated cells is 74 for *YAP1*Δ and 84 for *YAP1*Δ HS. Unless otherwise stated, data in graphs are mean ± SEM from three biological and three technical replicates. ***P < 0.001; **P < 0.01; *P < 0.05 (ANOVA plus post hoc).

Previous research has identified Yap1p as the transcription factor critical for the oxidative stress response in yeast, acting through upregulation of key enzymes of glutathione synthesis [27]. Therefore, we tested the Yap1p involvement in the glutathione recycling using a YAP1-deficient strain (*YAP1*Δ*). YAP1*Δ strain underwent an increase in the oxygen consumption (Figure 4A), as well as an almost 2-fold increase in the levels of mitochondrial superoxide during HS (Figure 4B). Transcript level analysis of the *YAP1*Δ strain during HS revealed metabolic changes consistent with the ones observed in WT strain: repression of glycolysis, along with increased transcript levels of the respiratory chain subunits, pentose phosphate pathway, and fatty acid oxidation enzymes (Figure 4C). Increased transcript levels of chaperones also revealed activation of the HSR (Figure 4C). In the context of antioxidant protection, superoxide dismutases (*SOD1* and *SOD2*) and the catalases (*CTA1* and *CTT1*) were upregulated (Figure 4C). However, there were no observed changes in levels of *GLR1* and *GSH1* compared with WT (Figure 4C). Consistent with these results, NADP^+^/NADPH ratio decreased during HS (Figure 4D), but the GSH level remained the same as at the OGT (Figure 4E). *YAP1*Δ strain was characterized by a median RLS of 11 generations at OGT and 9 generation at HS; its maximum RLS was 15 and 12 generations, at OGT and HS, respectively (Figure 4F). Therefore, the strain lacking the Yap1p transcription factor displayed a shortened RLS at OGT, and underwent a further decrease at HS, speaking in favor of importance of glutathione synthesis in the HS-induced longevity.

### TORC1 inactivation is necessary for heat shock-induced increase in respiration

**Figure 5:**
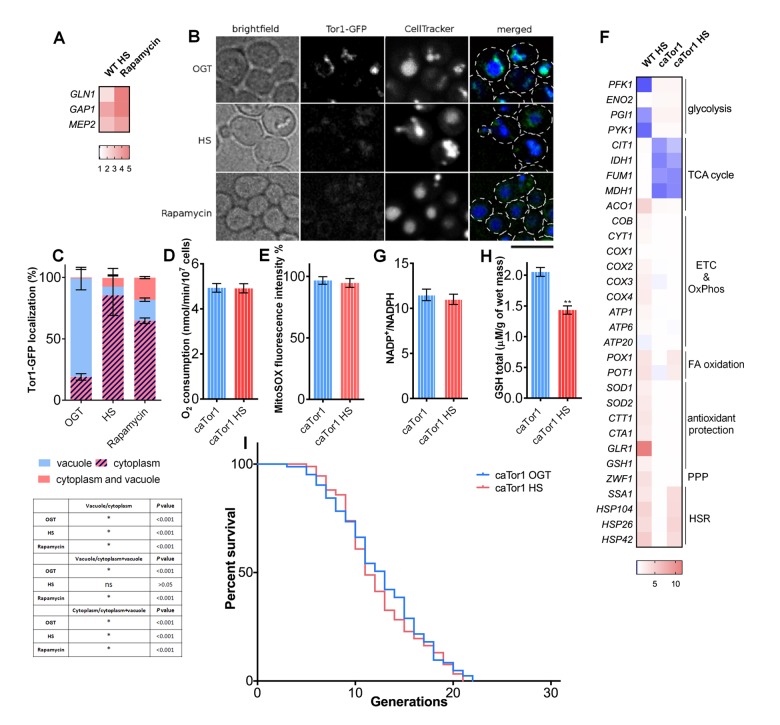
**TORC1 inactivation is essential for heat shock-induced activation of respiratory activity.** (**A**) qPCR measurements of expression levels of TORC1 transcriptional targets revealed their upregulation during heat shock, consistent with TORC1 inhibition using rapamycin. (**B**) Imaging using spinning disc confocal fluorescence microscopy showed deactivation of TORC1 by its displacement from the vacuolar membrane into distinct cytosolic puncta during heat shock and during rapamycin treatment. The black bar represents 5μm (**C**) Quantification of the cell fractions with differential localization of Tor1-GFP. Data on the graph are mean ± SD from three independent cultures. A minimum of 200 cells was quantified for each condition. *P* values calculated using multiple t-tests for each condition and shown in the table. *P <0.001. (**D**) Oxygen consumption remained unaffected during heat shock in the strain with constitutively active Tor1 kinase (**E**) Mitochondrially produced superoxide (measured by FACS as MitoSOX fluorescence in 10000 cells) was unchanged during heat shock in the strain with constitutively active Tor1 kinase. The results are presented as the percentage of MitoSOX fluorescence detected in WT strain at optimal growth temperature. (**F**) qPCR measurement of gene expression levels showed that the strain with constitutively active Tor1 kinase exhibited slight increase in levels of glycolytic enzymes and downregulation of TCA cycle compared with WT. (**G**) NADP^+^/NADPH ratio was not changed during heat shock in the caTor1 strain. (**H**) GSH levels were decreased during heat shock in the caTor1 strain. (**I**) Mean and maximum replicative lifespan (RLS) remain unchanged in caTor1 strain under heat shock. Control cells were kept constantly at 30^o^C. The number of curated cells is 83 for caTor1 and 92 for caTor1 HS. *P* value is >0.05 (Mantel-Cox). Unless otherwise stated, data in graphs are mean ± SEM from three biological and three technical replicates. ***P < 0.001; **P < 0.01; *P < 0.05 (ANOVA plus post hoc).

In order to gain insight into the mechanism of respiration activation during HS, we tested the involvement of TORC1 complex, previously shown to impact respiratory activity in yeast [28]. As reliable antibodies for phosphorylated Sch9p, the target of the TORC1 complex, are not available, we used the approach employed by Beck et al., that involved measuring transcriptional targets downstream of TORC1, which were shown to be upregulated upon TORC1 inhibition [29]. Increased transcript levels of *GLN1, MEP2*, and *GAP1* suggest that TORC1 was inhibited during HS, with a similar trend being observed after control treatment with rapamycin, an inhibitor of TORC1 (Figure 5A). This result was further supported by live cell imaging of yeast cells bearing GFP tagged Tor1p kinase (Tor1-GFP). Live cell imaging shows Tor1-GFP localized at the vacuole membrane (stained with CellTracker Blue) during OGT conditions, from where it relocated to the cytoplasm during HS, forming granules (Figure 5B) [30]. More specifically, at OGT, around 80% of the cells had Tor1-GFP localized to the vacuolar membrane (Figure 5B, Figure 5C). After 3 hours of HS this proportion of cells decreased to 7%, consistent with TORC1 deactivation: rapamycin treatment caused this proportion of cells to decrease to 17% (Figure 5B, Figure 5C). To test if TORC1 inhibition is required for the respiration increase during HS, we used a strain carrying an A1957V substitution within the Tor1p kinase rapamycin binding domain, making it constitutively active (caTor1) [20,31]. In the caTor1background, a 3 hour HS did not trigger an increase in oxygen consumption (Figure 5D), nor did it significantly influence mitochondrial superoxide levels (Figure 5E). qPCR measurement of gene transcript levels revealed already known changes in the metabolic activity characteristic for this strain already at optimal growth temperature [20]: slight increase in the expression levels of genes encoding for glycolytic enzymes and a decline for the TCA cycle enzymes (Figure 5F). However, at HS, respiratory chain, TCA cycle, glycolysis, and the antioxidant protection did not undergo any changes in gene expression levels (Figure 5F). The only changes that caTor1 strain exhibited at HS were the upregulation of *POX1* and *POT1*, regulatory enzymes of the fatty acid oxidation, as well as the upregulation of the heat shock response chaperones (Figure 5F). No changes could be observed in the NADP^+^/NADPH ratio during HS in caTor1 strain (Figure 5G), or in the GSH levels (Figure 5H). Finally, HS induced RLS extension was not observed in the caTor1 strain; median RLS for both conditions was 13 generations, while maximum RLS was 21 generations (Figure 5I).

The exact mechanism through which TORC1 is deactivated during HS will be subject to further study. However, our results so far present sufficient evidence that inactivation of TORC1 is indeed necessary for respiration increase during HS, as well as its downstream consequences; specifically, mild, transient HS mediated RLS extension. Furthermore, the transcript level analysis of the caTor1 strain shows that the repression of glycolysis is dependent on TORC1 deactivation under HS, and is parallel to and independent from the activation of respiration.

## DISCUSSION

Hormetic lifespan extension is a consequence of transient exposure to a low dose of a stressor such as heat, but the exact mechanisms that give rise to this effect are complex and not fully understood. It has been previously suggested that HS-induced protein misfolding triggers the activation of cell defense mechanisms, ultimately leading to lifespan extension [14, 15]. While several HS proteins are induced in yeast in response to heat stress, Hsp104p disaggregase has been proven essential for the HS-induced RLS extension [8, 32, 33], thus underscoring the importance of proper management of misfolded and aggregated proteins to survival during stress. Recently, another major contribution to reduced mortality under HS has been revealed: the opening of the diffusion barrier in the nuclear envelope promotes the heat stress-induced longevity in yeast, likely by enabling the damaged cell components to diffuse from the mother into the daughter cells when grown at 37°C [12]. Together, these observations show that the stress response is tightly controlled and adapted for the severity and duration, as well as the type of the stressor, as opposed to simply being a consequence of massive promotion of proteostasis by increased transcription of heat shock proteins alone. Notwithstanding, due to this complexity, one cannot exclude the existence of other mechanisms and pathways involved in stress survival, nor the interconnections between them.

**Figure 6:**
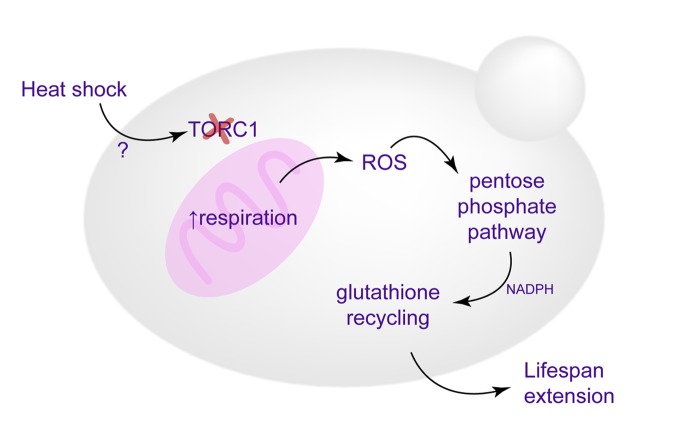
**Schematic representation of the proposed mechanisms culminating in the replicative lifespan extension.** Enhanced respiration, triggered by TORC1 inactivation, leads to increased superoxide production, which causes activation of antioxidant defenses and redox homeostasis maintenance (glutathione recycling), essential for heat-induced longevity.

Our results suggest that oxidative stress response, activated during HS as a reaction to enhanced respiratory activity and mitochondrial superoxide production, is essential to the HS mediated lifespan extension, as well as improved redox homeostasis (Figure 6). This conclusion is supported by several observations. First, the switch from fermentative to respiratory growth at HS was essential for the RLS extension (petite and *HAP4*Δ strains failed to extend RLS upon HS exposure). More importantly, as superoxide produced during enhanced respiratory activity is neutralized by the increased levels of Sod2p, the oe *SOD2* strain does not have extended RLS despite exhibiting the same switch from glycolysis to respiratory growth as WT. Furthermore, we find that redox homeostasis maintenance is epistatic to the enhanced respiratory activity under the conditions of mild HS in the WT strain. In the strain overexpressing *SOD2*, thereby suppressing the mitochondrial superoxide, the pentose phosphate pathway activation and consequent increase in NADPH and GSH levels were not observed, thus strengthening the conclusion that superoxide triggers the response ultimately leading to RLS extension. However, despite compelling evidence provided by the oe *SOD2* strain, based on the available results we cannot conclude that specifically superoxide is responsible for triggering the detected downstream events. Due to upregulation of Ctt1p and Cta1p we are not able to exclude the increase in the levels of hydrogen peroxide, H_2_O_2_, usually detoxified by glutathione and catalases. Therefore, we can conclude that compounded response of superoxide and hydrogen peroxide elicits the described changes and culminates in the RLS extension. The interplay between oxidative stress and HS has already been discussed in the context of its relevance in the survival of the severe HS [27], while our results underscore its importance in the heat induced hormesis. It should be emphasized that at this point we cannot comment on the long term benefits of the metabolic reprogramming during mild HS, as we did not investigate the sustainability of the observed metabolic changes in the mother cells that underwent HS. Whether the metabolic changes persist when the mother cells enter old reproductive age is an issue that will be addressed in future studies.

Here we find that during HS, cells undergo a fundamental change from consumption of glucose via glycolysis to respiration, usually characteristic of post-diauxic shift metabolism triggered by exhaustion of glucose from the medium. In this case however, yeast switch to respiratory metabolism during HS exposure in early exponential growth phase in a TORC1-dependent manner, despite glucose being abundant. It has previously been reported that, during HS, TORC1 is sequestered into stress granules, rendering it inactive [30]. We show that even mild and transient exposure to heat stress is sufficient to inhibit TORC1 activity by its sequestration into distinct foci. This is another example of TORC1-inactivation promoting longevity, a widely known phenomenon and one of the best studied lifespan extending treatments [34–37]. Although the molecular mechanisms of TORC1 inactivation during HS remain to be elucidated, it appears to be the pivotal event without which neither activation of respiration or the RLS extension cannot occur following HS (Figure 6), as demonstrated by the experiments performed with the strain carrying constitutively active Tor1p. Previous research has also reported on the importance of adaptive response to superoxide for chronological lifespan (CLS) extension observed in the yeast upon inhibition of TOR signaling, congruent with the results presented here. Sod2p has been shown to act downstream of the Sch9p, one of the targets of the TORC1 complex, and has been proven essential for the CLS extension observed in the *SCH9*Δ strain [38]. Consistent with our results, TORC1 inhibition (deletion of Tor1p or treatment with rapamycin) was shown to activate respiratory activity, thus increasing mitochondrial superoxide production and extending CLS in budding yeast [39–41].

Further, we uncovered an essential role of the PPP activation during HS as a source of NADPH used for glutathione reduction, the most distal event detected here that is responsible for the HS-mediated RLS extension (Figure 6). Dependence on glucose metabolism through the PPP has been proven important in neurons in the context of protection against superoxide produced by oxidative phosphorylation. In that context, production of NADPH via the oxidative branch of the PPP is indispensable due to its role as an electron donor in reduction of oxidized glutathione [42]. Redirection of glucose from glycolysis towards the PPP involves inactivation of glycolytic enzyme GAPDH and has previously been reported crucial in counteracting oxidative stress [43], and here we demonstrate its key role in the context of HS mediated lifespan extension.

Moreover, the interconnection between aerobic lifestyle and glutathione levels has previously been addressed. Several reports have shown that yeast is unable to grow on non-fermentable carbon sources in the absence of glutathione synthetase, Gsh1p (*GSH1*) [44,45]. Moreover, the role of glutathione in the cell survival during severe HS in yeast (41^o^C, 1 hour) has been investigated. Consistent with our results, GSH levels are shown to increase during severe HS in a Yap1p dependent manner [27] and provide an important contribution to cell survival of severe HS. The GSH increase is believed to protect the mitochondrial DNA from harmful mutations and is essential for survival of severe HS [27,45]. Our results extend the utility of oxidative stress response activated due to increased respiration past the one of enabling bare survival during severe stress, to being key to lifespan extension following mild and transient heat stress.

Together, these observations demonstrate that hormetic stress response is greater than the sum of its parts; while the relevant pathways are generally known and individually well studied, they often act together in complex and synergistic manner. Our results suggest that the cellular response to HS encompasses not only the response to heat-induced protein misfolding, but also the responses to oxidative stress that include antioxidant defenses supported by metabolic reprogramming. A better understanding of how hormetic effects come about after cells survive different types of stress does not only strengthen our understanding of cellular processes but also opens up new directions in hormesis mimetics research.

## MATERIALS AND METHODS

### Strains and growth conditions

Wild type *Saccharomyces cerevisiae* Y258 was used. WT Y258 and the Sod2p overexpressing (oe *SOD2*) strains were purchased from Thermo Scientific (Dharmacon), and deletion mutants were constructed using a homologous recombination based procedure whereby the target gene was replaced by a hygromycin B resistance cassette [46]. Hygromycin B resistance cassette was amplified from pAG32 plasmid (Addgene). Oligonucleotides used for gene deletions are listed in the Table S2. Petite mutant strains were constructed following a standard protocol [47]. Y258 and petite strains were grown on YPD medium with 2% (w/v) glucose, deletion mutants on YPD medium with 2% (w/v) glucose and 100 μg/mL hygromycin B (Sigma), and oe *SOD2* strain in uracil drop-out (-URA) medium with 2% (w/v) glucose, at 30^o^C with shaking. Expression from the plasmid was induced by 2% galactose using the following procedure: overnight culture was diluted 100x and grown until mid-exponential phase. Then, the cells were pelleted by a 5-min centrifugation at 4000 g and resuspended in -URA medium without glucose, supplemented with 2% galactose. Empty vector control is always treated in the same way.

HS experiments were performed at 34^o^C with shaking for 3 hours starting from early exponential phase, OD 0.2-0.3, while the control cells were kept at 30^o^C. For pharmacological treatments the following final concentrations were used: 220 nM rapamycin, and 10 μM carbonyl cyanide m-chlorophenyl hydrazine (CCCP). The rapamycin treatment was performed for 90 minutes starting in the mid-exponential growth stage, OD 0.5-0.6. The cells were exposed to CCCP during HS, and at optimal growth temperature for control, starting in early exponential phase (OD 0.2-0.3) and lasting 3 hours. Unless otherwise stated, the cells were harvested by 5-minute centrifugation at 3500 × g, washed and treated accordingly.

### Replicative lifespan measurement

Replicative lifespan (RLS) for all strains was determined by micromanipulation. RLS measurement involves counting the number of daughters produced by individual mother cells. Control cells were incubated at 30°C on YPD plates for the duration of the experiment. HS was performed by placing the YPD plates at 34^o^C for 3 hours at the cell replicative age of 1-3 generations. For the RLS measurement of the oe *SOD2* strain, -URA plates were used. Briefly, using a microdissection apparatus equipped microscope suitable for yeast (Singer Instruments), cells were transferred to defined places on the agar plates and virgin daughter cells were collected. Each cell was monitored continuously over several days every 60-90 minutes until all mother cells stopped budding. A total number of daughter cells was noted for each mother cell.

### GSH extraction and measurement

Cells were collected, washed and wet weight of the cell pellets was measured. Then, cell wall was lysed in 10mM PBS supplemented with zymolyase (0.06 U/µL). Solution containing spheroplasts was centrifuged at 3500 x g, and the pellet was further treated with spheroplast lysis buffer (1mL buffer per 0.5 g of cell pellet; 0.6 M sorbitol, 10 mM Tris-HCl (pH 7.4), 1 mM PMSF)*.* The solution was kept on ice for 30 minutes with occasional vigorous vortexing, and centrifuged at 14,000 x g. Pellet was discarded. In order to avoid the detection of thiol groups from proteins, proteins were precipitated using trichloracetic acid (TCA), where 1 volume of 100% TCA was mixed with 4 volumes of cellular extract followed by a 10-minute incubation at 4^o^C. Protein precipitate was pelleted by centrifugation at 10,000 x g for 5 minutes. The supernatant was collected and used for the measurement of GSH by the method of Ellman [48] 2mM 5,5'-Dithio-bis(2-nitrobenzoic acid) (DTNB, Ellman’s reagent, Sigma) stock solution was prepared and stored at 4^o^C. DTNB working solution was prepared by pre-mixing 50 μL of the DTNB stock, 100 μL of 1M Tris pH 8.0 and 840 μL of water, which is then mixed with 10 μL of the sample, followed by a 5-minute incubation at room temperature. Absorbance is measured at 412 nm. Molarity of SH groups is then calculated by dividing the absorbance with the molar extinction coefficient, 13600M^-1^ cm^-1^.

## Growth curves

Overnight cultures grown at 30°C were diluted 50x in YPD with 2% glucose, and 200 µL were aliquoted in triplicate in U-bottom transparent 96 well plates. Plates were incubated in a temperature controlled Tecan Infinite F200 instrument with shaking, and OD measurement was taken every 15 minutes to construct the growth curves. Generation times were extrapolated from the exponential growth phase of the curve using the following formula, where N_0_ is the beginning OD value, N_t_ is the ending OD value, and t is the time between N_0_ and N_t_:

$$\frac{log10(\frac{Nt}{N0})}{o.3}=g\;;\frac{t}{g}=doubling\;time$$

### Respiration measurement

Oxygen uptake was monitored polarographically with an oxygraph equipped with a Clark-type electrode (Oxygraph, Hansatech, Norfolk, UK). Cells were harvested during the exponential growth phase, spun and resuspended in appropriate medium at the density of 30 × 10^6^ cells/mL. 500 µl of culture was transferred to an airtight 1.5 mL oxygraph chamber. Cells were assayed in conditions closely similar to the ones in a flask culture (30°C and stirring). Oxygen content was monitored for at least 4 minutes. To ensure the oxygen consumption observed was due to the mitochondrial activity, complex III inhibitor antimycin (final concentration 10 μg/mL) was routinely added to the cultures and compared to the rate observed without antimycin.

### Flow cytometry – ROS level measurements

For the mitochondrial superoxide measurement, cells were incubated in the dark with 3 μM MitoSOX Red (Thermo Scientific) for 10 minutes at 37°C with shaking. For the total ROS level measurement, cells were incubated in the dark with 10 μg/μL 2’,7’-dichlorofluorescein diacetate (H2DCFDA, Sigma) for 120 min at 37°C. Subsequently, all samples analyzed on FACSCalibur flow cytometer. Fluorescence of both dyes was measured in 10,000 cells using excitation at 488 nm and monitoring emission at 635 nm. The collected data was analyzed using FlowJo software version 7.2.5 for Microsoft (TreeStar, San Carlos, CA, USA) to determine the mean green fluorescence intensity after each treatment. The results are expressed as the mean fluorescence of the 10,000 cells. MitoSOX as well as H2DCFDA fluorescence of all strains is expressed as the percentage of the WT fluorescence at optimal growth temperature.

### NADP^+^/NADPH ratio measurement

NADP^+^/NADPH ratio was measured using the NADP^+^/NADPH Quantification Kit from Sigma (MAK038) according to the manufacturer's instructions. Briefly, cells were harvested as previously described and washed using cold 10 mM PBS pH 7.4, followed by a 1 hour zymolyase treatment at 37^o^C. Spheroplasts were harvested by centrifugation at 3000 rpm for 5 minutes and treated with NADP^+^/NADPH extraction buffer supplied in the kit for 10 minutes on ice. Samples were then centrifuged at 10000 × g for 10 minutes and the supernatant was collected. Protein concentration was measured in the supernatant and used for normalization of NADP^+^/NADPH ratios. Aliquots of the extracted samples were stored for measurement to give the levels of NADP_total_. In the rest of the extracted samples, to detect NADPH, NADP^+^ is decomposed by heating the extracted samples to 60°C for 30 minutes in a water bath, followed by cooling on ice. Under these conditions, all NADP^+^ is decomposed leaving only NADPH. Standards and samples were distributed into a 96-well plate and exposed to a master reaction mix followed by a 5-minute incubation at room temperature and the addition of NADPH developer, supplied in the kit. The absorbance is then read at 450 nm. The NADP^+^/NADPH ratio is calculated according to the following equation:

ratio= (NADP_total_-NADPH)/NADPH

### Vacuole staining

Yeast cultures were grown and treated as described above for the OGT control, HS treated culture and the treatment with rapamycin. CellTracker Blue CMAC (4-amino-4chloromethylcoumarin; Invitrogen) dye was added directly to the culture medium (0.1 mM final concentration) to the growing cultures and incubated for 30 minutes. Immediately after staining cells were collected and imaged as described below.

### Live cell imaging

### *Slide preparation*

Microscope slides were prepared as follows: 200 μL of YPD media containing 2% agarose was placed on a preheated (30^o^C for OGT and 34^o^C for HS) microscope slide, covered with a cover slip, and cooled before applying yeast cells directly onto the YPD-agarose pad to obtain a monolayer. Once dry, the cover slip was placed back and sealed. The slide was mounted on temperature-controlled Nikon Ti-E Eclipse inverted/UltraVIEW VoX (Perkin Elmer) spinning disc confocal setup, driven by Volocity software (version 6.3; Perkin Elmer). Images were recorded through 60xCFI PlanApo VC oil objective (NA 1.4) using coherent solid state 488 nm/50 mW diode laser with DPSS module, and 1000x1000 pixels 14 bit Hamamatsu (C9100-50) electron-multiplied, charge-coupled device (EMCCD). Unless specified otherwise, images were processed using Image J software.

### *Tor1p localization analysis*

Untreated control strain with Tor1-GFP fusion (obtained as a gift from Prof. Tatsuya Maeda) as well as Tor1-GFP strains treated with 220 nM rapamycin or HS (described elsewhere) were centrifuged at 4000 × g for 3 minutes, resuspended in ≈50 μL YPD, and placed on agarose pads and imaged as described above. Images were analyzed using ImageJ software and manually scoring fractions of cells displaying distinct Tor1-GFP localization. Cells were scored as having completely vacuolar, completely cytosolic, or Tor1-GFP localization in both compartments.

### *Mitochondrial morphology*

Mitochondrial morphology was monitored using the MitoLoc plasmid (obtained as a gift from Prof. Markus Ralser) [49]. Each studied yeast strain was transformed according to the described protocol [50] with the only difference that the cells were incubated with the plasmid overnight at room temperature. The exposure time was 100 ms for GFP and 5–10% laser intensity was used. Approximately 500 cells of each condition were examined. Images were analyzed using ImageJ software with the MitoLoc plugin.

### Statistical analysis

Unless otherwise stated, data in graphs are mean ± SEM from three biological and three technical replicates. ***P < 0.001; **P < 0.01; *P < 0.05 (ANOVA plus post hoc or log-rank (Mantel–Cox) test.

### RNA extraction

Total RNA was isolated from yeast cells following the procedure of the NucleoSpin RNA kit (Macherey & Nagel) for up to 3 × 10^8^ yeast cells, which dictates incubation with 50-100 U of zymolyase for 1 hour at 30°C. The quality of resulting total mRNA was tested on 1% agarose gels.

### Quantitative real-time PCR

cDNA was synthesized from 1000ng of total RNA using iScript^TM^ cDNA Synthesis Kit (Biorad). The resulting cDNA was diluted 100×, mixed with primer pairs for each gene and SYBRgreen (BioRad). All primer pairs were designed using Oligo Calc: Oligonucleotide Properties Calculator to have a melting temperature of 60°C and their sequences are given in Table S2. The qPCR reaction was run on a QuantFlexStudio 6 (Life Technologies) using 40 cycles, after which the melting curves for each well were determined. qPCR differential expression was estimated with EasyqpcR (https://bioconductor.org/packages/release/bioc/html/EasyqpcR.html) using the methods from [51]. Final fold change values were estimated relative to the *UBC6* gene in the control strain replicates.

### RNA Sequencing

### *Sequence mapping*

Sequenced reads (Illumina 50bp single-end) were mapped to the SacCer3 reference genome (April 2001 revision,http://www.ncbi.nlm.nih.gov/assembly/285498/, genome obtained from the UCSC Genome Browser sitehttp://genome.ucsc.edu/ [52],) using the STAR aligner (version 2.3.0e, [53]). Reference transcripts were obtained from the Ensembl yeast genome resource in GTF format.

### *Read counting*

Raw read counts were determined from the overlaps with the Ensembl yeast genes using the htseq-count version 0.6.0. from the HTSeq framework [54], with the following parameters: -a 10 –s no.

### *Differential expression analysis*

Raw reads were analyzed for differential expression with the DeSeq2 package [55] within the Bioconductor framework [56] under R statistical package (R Core Team), by previously filtering out the outlier replicates with PCA analysis. Selected genes were visualized in a heatmap by using log2(FC) values. Conditions with adjusted p-value lower than 0.1 were crossed out as not significant (labeled with a gray X sign).

## Supplementary Material

Supplementary Figures

Table S1

Table S2
