# Supplementary material for: Heat-induced longevity in budding yeast requires respiratory metabolism and glutathione recycling
Source: Aging (Albany NY). 2018 Sep 17;10(9):2407–27. doi: 10.18632/aging.101560 (PMC6188503; doi:10.18632/aging.101560)
Supplement: Supplementary Figures [file aging-10-101560-s001.pdf]

SUPPLEMENTARY MATERIAL

SUPPLEMENTARY FIGURES

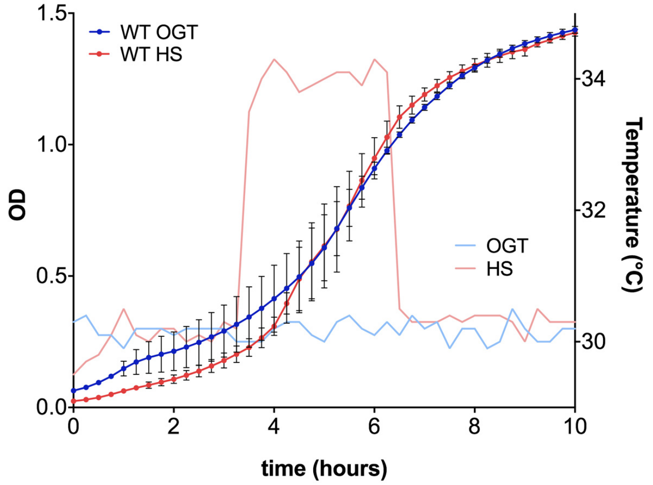

Figure S1. WT strain of budding yeast is characterized by accelerated growth during 3 hour heat shock at 34°C. Doubling time is accelerated in WT budding yeast that undergoes a 3 hour heat shock at 34°C early in their life (onset of exponential growth phase, corresponding to 1-3 generations of age), from 120 minutes (± 6 minutes) when grown constantly at 30°C, to 72 minutes (± 4 minutes) after the 3 hour heat shock. Doubling time was calculated from the exponential phase of the growth curves. Growth curves are derived from biological and technical replicates; error bars represent SD.

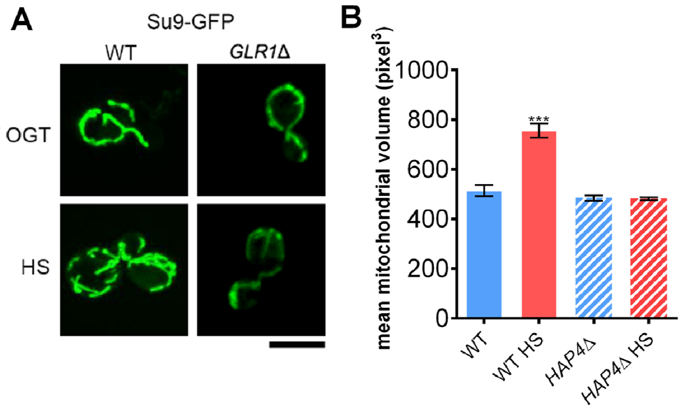

Figure S2. Heat shock leads to increased mitochondrial volume. (**A**) The images display representative examples of mitochondrial morphology of WT and *HAP4Δ* at optimal growth temperature and immediately following 3 hour heat shock. Imaging was performed using spinning disc confocal fluorescence microscopy. OGT stands for optimal growth temperature and HS for heat shock. The black bar represents 5 μm. (**B**) Quantification of the mitochondrial volume was performed using the MitoLoc plugin in ImageJ. More than 300 cells from two biological replicates were analyzed and the mean value of mitochondrial volume ± SEM is displayed on the graph.

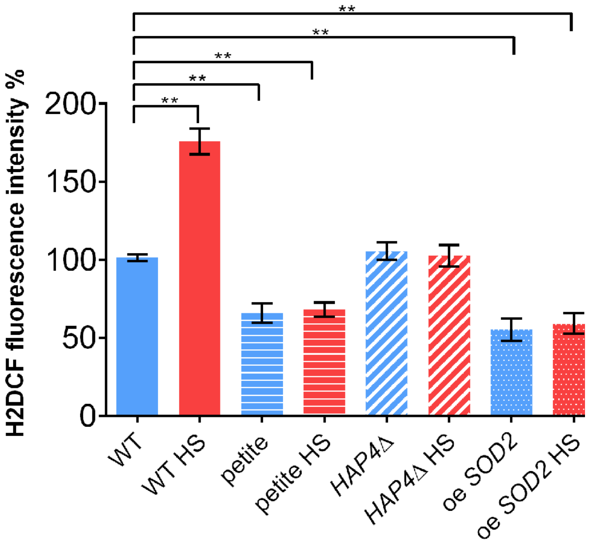

Figure S3. ROS increases following heat shock in WT but not in petite or HAP4Δ strains. Data on the graph are mean ± SD from three biological and three technical replicates. P values were calculated using ANOVA plus post hoc. ***P < 0.001; **P < 0.01; *P < 0.05.

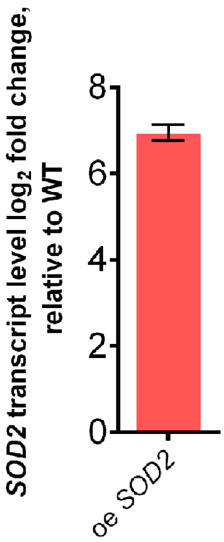

Figure S4. SOD2 expression level in oe SOD2 strain. Data on the graph are mean ± SEM from three biological and three technical replicates.

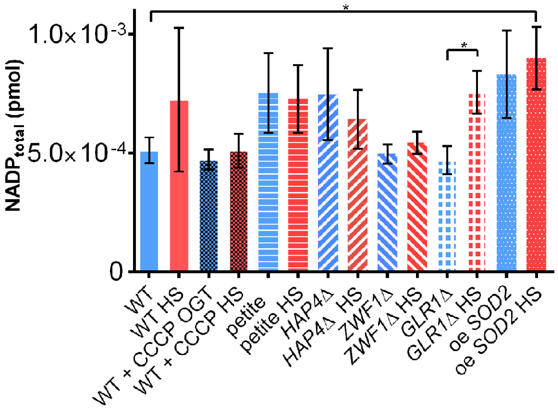

Figure S5. The total amount of $NADP^+$ does not vary significantly in any of the studied strain. Data on the graph are mean ± SEM from three biological and three technical replicates. *P* values were calculated using ANOVA plus post hoc. ****P* < 0.001; ***P* < 0.01; **P* < 0.05.

SUPPLEMENTARY TABLES

Please browse Full Text version to see the data of Supplementary Tables:

Table S1. The summary of the RNA sequencing results with the data for each strain and condition organized in separate tabs.

Table S2. The list of primers used for the measurement of the gene transcript levels by qPCR, as well as primers used for cloning, listed in separate tabs.
